# Glucosinolates from Broccoli By-Products Obtained by Pressurized Liquid Extraction Exert Anti-Inflammatory Activity on Non-Malignant Colonic Myofibroblasts

**DOI:** 10.3390/plants14111700

**Published:** 2025-06-03

**Authors:** María Borja-Martínez, Jesús Lozano-Sánchez, Rosa Quirantes-Piné, Lorena Almagro, María A Pedreño, Ana B. Sabater-Jara

**Affiliations:** 1 Department of Plant Biology, Faculty of Biology, University of Murcia, Campus de Espinardo, 30100 Murcia, Spain; maria.borja@um.es (M.B.-M.); lorena.almagro@um.es (L.A.); mpedreno@um.es (M.A.P.); 2 Department of Food Science and Nutrition, University of Granada, Campus of Cartuja, 18071 Granada, Spain; jesusls@ugr.es; 3 Department of Analytical Chemistry, Faculty of Sciences, University of Granada, Campus Fuentenueva s/n, 18071 Granada, Spain; rquirantes@ugr.es

**Keywords:** broccoli by-products, CCD-18Co myofibroblasts, glucosinolates, inflammatory bowel disease, lipopolysaccharide, pressurized liquid extraction, proinflammatory cytokines, reactive oxygen species

## Abstract

Broccoli agro-industries generate a significant amount of waste, which leads to both environmental and economic problems. These by-products are typically discarded, despite being a valuable source of bioactive compounds, including glucosinolates (GSLs), which can modulate oxidant and inflammatory mediators, exerting anti-inflammatory properties. A crucial challenge in the exploitation of broccoli agro-industrial by-products is the development of sustainable and green extraction technologies. In this work, pressurized liquid extraction (PLE) based on response surface methodology (RSM) has been developed and optimized for the GSLs’ extraction, with the aim to evaluate the potential protective mechanisms triggered by GSLs-enriched extracts from broccoli by-products in CCD-18Co myofibroblasts exposed to lipopolysaccharide (LPS). The results obtained showed that the PLE is an efficient and environmentally sustainable alternative procedure for extracting GSLs from broccoli by-products. Furthermore, the GSLs-enriched extract obtained through PLE exhibited antioxidant and anti-inflammatory properties in LPS-stimulated cells, being able to attenuate the expression of some proinflammatory markers (IL-1β, IL-6, IL-8, and TNF-α). Therefore, these compounds could serve as potential nutraceutical agents for the prevention and mitigation of oxidative and inflammatory processes related to intestinal bowel diseases, while also promoting the valorization of these by-products.

## 1. Introduction

Nowadays, Western diets are rich in fat and cholesterol, which are largely considered responsible for obesity and metabolic syndrome. These factors have also been shown to increase the permeability of the intestinal barrier [1]. Given the high incidence of these pathologies in the population, one of the most prominent conditions today is inflammatory bowel disease (IBD), a chronic condition that includes Crohn’s disease and ulcerative colitis, both characterized by inflammation of the intestinal tract’s mucosa [2,3]. Some inflammatory mediators that are involved in the pathogenesis of IBD are proinflammatory cytokines, particularly IL-6, TNF-α, and IL-1β [4,5]. These cytokines are associated with the initiation and progression of IBD, resulting from the recruitment of monocytes and activated macrophages, which in turn generate chemical mediators that mediate inflammation, such as prostaglandin E2, through the induction of cyclooxygenase-2 (COX-2) isoenzyme. Thus, an excess of inflammatory mediators in the colon tissue can lead to edema, ulcers, and even carcinogenesis, so effective regulation of the secretion of these inflammatory factors is decisive in the treatment of IBD [6].

In this scenario, it is important to highlight the structure and function of the lipopolysaccharide (LPS), an endotoxin that constitutes the main component of the outer surface of most Gram-negative bacteria and is one of the most potent stimuli of inflammation. Thus, when LPS is released from the membrane of the bacteria that colonize the colon, it can bind to receptors on the membrane of the epithelial cells, triggering an inflammatory response in which mediators such as TNF-α and proinflammatory cytokines are involved, including IL-1α, IL-1β, IL-6, IL-8, and IL-10 [2,7,8]. Furthermore, when the epithelial barrier is damaged, LPS can also enter the bloodstream, leading to endotoxemia and initiating a systemic inflammatory response. Once in the blood, LPS interacts with the surface receptors of the immune system’s cells, triggering an inflammatory response that activates the factor NF-κB, which in turn promotes an increase in the transcription of proinflammatory cytokines such as TNFα, IL-1β, and IL-6. That means that, in case of dysfunction of the epithelial barrier of the intestinal mucosa, LPS can cause other pathologies related to inflammation in other parts of the body [1,2,9,10]. Moreover, when the continued release of LPS in the intestine, combined with the predominance of diets rich in fat and cholesterol, becomes a chronic situation, the epithelial barrier of the intestine atrophies since the junctions between the cells of the epithelial mucosa are damaged. This dysfunction of the intestinal barrier is considered the main cause of IBD [2]. In this sense, compounds capable of reversing this situation of chronic inflammation in the colon have been studied as potential pharmacological ingredients that can relieve the symptoms of these illnesses.

There are numerous studies that inversely relate the consumption of fruits and vegetables, especially dark green ones, specifically brassicas, which are rich in glucosinolates (GSLs), and the risk of developing colon cancer or diseases such as IBD [11]. Broccoli (*Brassica oleracea* var. italica), a member of the cruciferous family, is a vegetable enriched in bioactive compounds, including GSLs, polyphenols, vitamins, minerals, carbohydrates, and fibers [12]. These compounds have a variety of health-promoting activities, including antioxidants, antibacterial, anticancer and anti-inflammatory activities [13,14]. Among them, GSLs, also known as thioglucosides, are nitrogen- and sulfur-containing anionic secondary metabolites found almost exclusively in plants of the Brassicaceae family, and they are responsible for most of the bioactive properties of these plants. GSLs and their hydrolysis products, mainly sulforaphane (SFN), have been described as promising anti-inflammatory agents that help to reduce IBD symptoms by suppressing the levels of proinflammatory cytokines and enhancing anti-inflammatory cytokine production [5,15].

On the other hand, many studies suggest a clear relationship between the intake of broccoli and the development and maintenance of an inflammatory state. Nevertheless, despite its numerous beneficial effects on human health, the main target of the edible portion (florets, tender stems, and sprouts) is food consumption, but this part constitutes only 15% of the entire plant, which generates a substantial amount of by-products, i.e., leaves and stems (60 to 75% of the total biomass) that are commercially underutilized and mostly discarded as waste during harvesting, and, in most cases, they are used as livestock feed [16,17] or for the production of compost for fertilizing crop fields [18,19]. In addition to the edible parts of broccoli, the agro-industrial by-products are also rich in bioactive compounds, including GSLs, which can be used as potential ingredients for food, cosmetics, and/or pharmaceutical industries [20]. However, a major challenge in utilizing these agro-industrial by-products is the development of sustainable and green extraction technologies [21]. In this regard, over the past ten years, extraction processes have focused on the search for environmentally friendly solvents and the development of increasingly green and sustainable extraction techniques to obtain high extraction yields with reduced cost while maintaining the structural integrity of the desired compound [22]. One technique that has gained the most popularity in recent years is the obtention of bioactive compounds using pressurized liquid extraction (PLE). This extraction method has been emerging as an alternative extraction technology due to its efficiency in terms of time, solvent use, and compound recovery, making it a safer and faster technique compared to conventional extraction (CE) techniques. PLE involves using water and other hydroalcoholic solvents, and it combines two main elements: (1) increased temperature and pressure, and (2) the interaction between the solvent and the matrix compounds [23]. This green technology has been applied to recover bioactives such as flavonoids, anthocyanins, phenolic acids and other polyphenols, saponins, or essential oils from a variety of plant sources, as well as GSLs from the Brassicaceae family [24,25,26].

Based on this background, we hypothesized that GSLs extracted from broccoli by-products using PLE can protect colon fibroblasts from LPS-induced inflammation by activating the signaling pathways that trigger inflammatory processes. Thus, to gain further insight into how GSLs contribute to protecting cells against intestinal inflammation, the present study aimed to evaluate the protective mechanisms triggered by GSLs-enriched extracts from broccoli by-products in CCD-18Co myofibroblasts exposed to LPS, and to elucidate the role of these compounds in preventing and mitigating oxidative and inflammatory processes and promote the valorization of these by-products.

## 2. Results

### 2.1. Optimization of the PLE Conditions to Obtain GSLs from Broccoli By-Products

To establish the optimal conditions to achieve the maximum recovery of GSLs (response variable) from broccoli by-products through PLE (GSLs-PLE), an experimental design based on a response surface methodology (RSM) was developed. The effect of (a) temperature (53–200 °C), (b) ethanol percentage (5–90%), and (c) time (3–22 min) on the GSL recovery from broccoli by-products was evaluated according to a central composite design 2^3^ (CCD) (Table 1). The experimental range for these independent variables was set based on previous literature support [20].

Once the experimental conditions were established, the GSLs content (response variable) present in the 16 conditions was quantified by HPLC-MS (Table 1). The results obtained indicated that the best conditions for extracting the highest amount of total GSLs were PLE-15, followed by PLE-16 and PLE-3. Under these conditions, 3649 ± 116, 2930 ± 94, and 2565 ± 81 μg GSLs g^−1^, respectively, were obtained. These experiments corresponded to those in which the extraction temperature was one of the lowest studied, 70 °C for PLE-15 and PLE-3 and 53 °C for PLE-16 (Table 1). The combination of low temperatures with medium–high percentages of ethanol could improve the recovery of the target compounds, as has been described below. On the other hand, those conditions with higher temperatures (185–200 °C), PLE-7, 9, 11, 13, and 14 were the ones where the least amount of GSLs were extracted. Despite that some of these experiments were developed at high ethanol percentages (PLE-11 and PLE-14), a significant GSLs content reduction was observed. This fact could be because of thermal degradation due to the extraction temperature. Regarding the extraction yield, as observed in Table 1, the values obtained ranged between 22.42% in the case of the PLE-10 extraction and 56.31% in the PLE-7. However, the highest GSLs content was obtained in those conditions in which the EY was lower, as observed in the PLE-15 condition (3649 ± 116 μg GSLs g^−1^; EY-27%).

**Figure 1:**
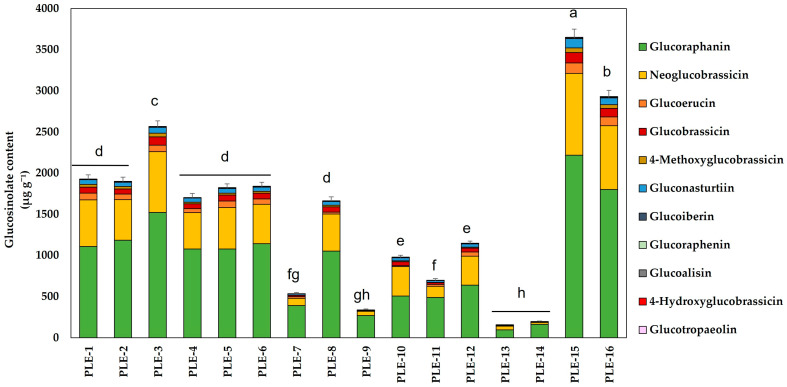
Total glucosinolates (GSLs, μg g^−1^ extract) obtained from broccoli by-products extracted by pressurized liquids (PLE). Data show the mean ± SD of three independent replicates. Different letters indicate statistically significant differences according to Tukey’s test (*p* < 0.05).

Regarding the individual content of GSLs (Figure 1), most of the GSLs were glucoraphanin (an aliphatic GSL precursor of SFN). This GSL accounted for an average of 64% of the total GSLs, with values reaching 1799 ± 99 μg g^−1^ (PLE-16). Following this, neoglucobrassicin accounted for an average of 25% of the total GSLs extracted. Together, these two GSLs represented nearly 90% of the total quantified, with the remaining GSLs found in significantly lower amounts compared to the two major GSLs already mentioned.

**Figure 2:**
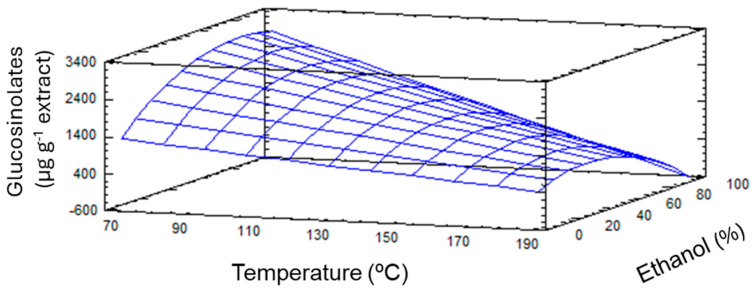
The 3D predicted response surface plots of the effects of temperature and percentage of ethanol on the glucosinolates content.

The experimental results obtained from the quantification of GSLs at different points of the experimental design were evaluated to assess the fit of the proposed CCD 2^3^ model, as well as the statistical significance of the effects of the independent variables. The fitting parameters for the proposed CCD 2^3^ model and the statistical significance of the independent variable effects are shown in Table 2. To facilitate the interpretation of the model, only the individual effects and the quadratic and interaction effects between variables with a significant impact are included in the table. This analysis revealed a high degree of correlation (*R*^2^ = 0.942) which explains the variance within the data, as this value evaluates the ability of the model to predict the behavior of the response variable. Furthermore, the lack of fit was not significant (*p*-value > 0.1), verifying the fitting quality of the model. Therefore, the model provided a good approximation to the experimental conditions (Figure 2), as confirmed by the parameters obtained by ANOVA analysis (Table 2).

The probability values (*p*-value) of the linear, quadratic, and interaction effects between the independent variables were analyzed. In this case, only temperature presented a significant individual effect (*p*-value of 0.0000) (Table 2). Regarding the percentage of ethanol used in the extraction, even though its linear effect did not show a significant effect on the value of the response variable, the interaction with temperature did result in a statistically significant impact on the GSLs content (*p*-value = 0.0059). These results could suggest a synergy between temperature and the percentage of ethanol used to maximize the production of GSLs, a conclusion like those obtained by other studies focused on the optimization of this process [27]. The quadratic effect of ethanol was also significant (*p*-value = 0.0298). Based on these results, the experimental design was adjusted to a quadratic polynomial model that was reduced to keep the terms of the independent variables and their significant effects for the response variable (Y, in this case μg GSLs g^−1^) (Equation (1)):

*Y* = 1831.28 − 7.6311*X*_1_ + 58.7085*X*_2_ − 0.207819*X*_1_*X*_2_ − 0.287463*X*_2_*X*_2_ (1)

Thus, the optimal conditions for the maximum recovery of GSLs predicted by the model were as follows: 53 °C and 83% ethanol. It should be taken into account that despite the fact that the extraction time did not exert a significant effect, the data processing and statistical analyses by the validated and fitted model provided 8 min as a reference value for this variable.

Under the optimized PLE conditions, the maximum amount of GSLs extracted was 4530 ± 132 μg g^−1^, which was even higher than that obtained with the most favorable condition of the experimental series, PLE-15 (3649 ± 116 μg g^−1^). Furthermore, as shown in Table 3, when comparing the predicted values by our model for the total content of GSLs based on Equation (1) with those obtained under optimal conditions, it was observed that, except for under PLE-13 and PLE-14 conditions, the variance of the data was acceptable, all being within the range of 0.6 to 10.3 (CV < 20%). In fact, the variance between the theoretical experimental data under optimal conditions was low (CV = 10 %), indicating a good reproducibility of the investigated system [28].

Once the optimal conditions for the GSLs extraction by PLE were established, the optimized extraction of GSLs from broccoli by-products was performed (Table 4). The extraction yield of this optimized procedure was 21%, similar to the yield obtained under the PLE-15 conditions (27%, Table 1), which produced the highest amount of GSLs (Figure 1). However, when comparing these results with those obtained with the conventional extraction (CE), we observed that with PLE, the total content of GSLs was two times lower than that obtained by CE (4530 ± 132 μg g^−1^ and 9015 ± 449 μg g^−1^, respectively) (Table 4). Despite this difference in GSLs content, it did not result in a significant reduction in EY, obtaining yields of 21 and 31% in PLE and CE, respectively. This difference could be explained by the conditions of both methods. It is likely that the 53 °C used for extracting these compounds by PLE is not sufficient to inactivate the myrosinase enzyme (compared to the 73 °C used in CE, which is 20 °C higher), and this enzyme breaks down GSLs into their isothiocyanates. This factor appeared to be the most influential for obtaining GSLs by PLE, as reflected in Table 2, with the *p*-value of temperature being 0.000. However, similar results were reported by some authors in the optimization of the GSLs extraction processes. In this sense, using an RSM model, Campos et al. [29] concluded that the optimal conditions for achieving the highest yield in the extraction of GSLs in hypocotyls of *Lepidium meyenii* were the use of 60% ethanol and a temperature of 47 °C, although in this case a longer extraction time of 90 min was used. Similar results were obtained by Bojorquez-Rodríguez et al. [27], who established the optimal conditions for GSLs extraction by PLE from broccoli sprouts in 50% ethanol and a temperature of 40 °C. In this scenario, it could be assumed that the combination of RSM with green and advanced extraction techniques, such as PLE, allows the recovery of bioactive compounds, expending less energy and time as well as low solvent consumptions, offering an alternative to apply relevant environmentally friendly methodologies for obtaining high quality extracts.

### 2.2. Cytotoxicity of the GSLs-Enriched Extract and LPS on the CCD-18Co Cell Line

**Figure 3:**
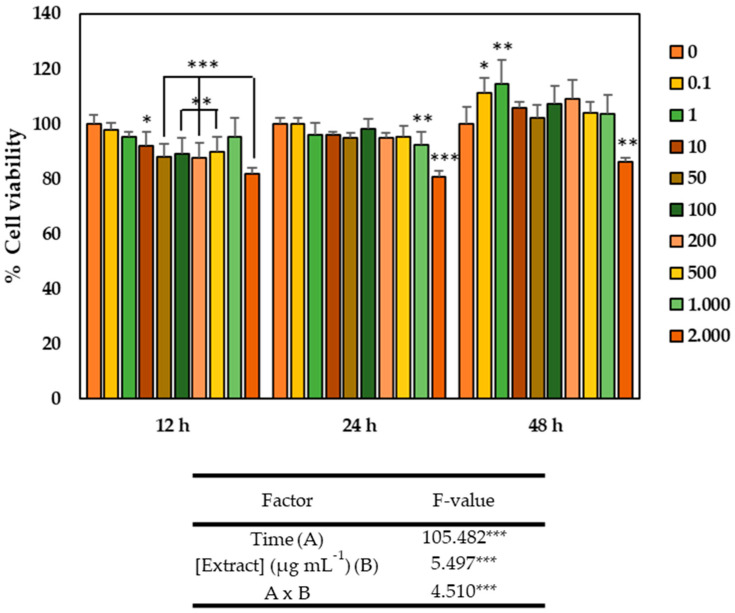
Viability of CCD-18Co cells after 12, 24, and 48 h of exposure to glucosinolates (GSLs) extracts (0–2000 µg mL^−1^) obtained by pressurized liquids (PLE) from Naxos broccoli by-products. Data show the mean ± SD of six independent replicates. Asterisks indicate significant differences: * *p* < 0.05; ** *p* < 0.01; *** *p* < 0.001.

The optimized GSLs-enriched extract obtained from broccoli by-products by PLE were used for cytotoxicity studies on the healthy colon myofibroblast cell line CCD-18Co to determine those concentrations of the extracts that caused an inhibitory effect on the cell growth. For this, the evaluation of the cytotoxic effect of different concentrations of the GSLs extract over time was carried out. As observed in Figure 3, the viability of the CCD-18Co cell line was significantly influenced by both the extract concentration and time, as well as by the interaction of both factors (*p*-value < 0.001). Thus, in terms of time, cell viability increased significantly, being maximum after 48 h. At this time, cells treated with different concentrations of GSLs extract showed higher viability than the control, except for the treatment with 2000 µg mL^−1^, which significantly reduced viability, while the two lowest concentrations studied (0.1 and 1 µg mL^−1^) were not only non-cytotoxic, but also significantly increased cell viability, reaching up to 111 and 114% viability, respectively.

On the other hand, LPS is considered one of the most potent stimulants of inflammation. Although several studies have demonstrated the anti-inflammatory activities of bioactive compounds from different plant-based foods, studies on the effects of broccoli GSLs on cell signaling pathways involved in intestinal inflammation are quite limited. Therefore, the aim of this study was to evaluate the effect of a GSLs-enriched extract obtained by PLE from broccoli by-products on some proinflammatory markers and signaling pathways involved in normal colonic CCD-18Co myofibroblast cells stimulated with LPS.

**Figure 4:**
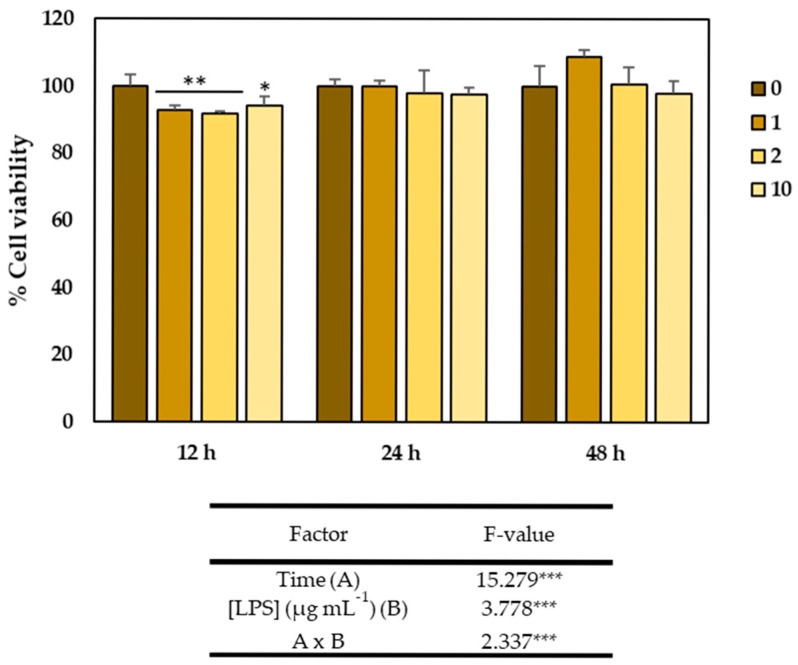
(A) Viability of CCD-18Co cells after 12, 24, and 48 h of exposure to different lipopolysaccharides (LPS) concentrations (0, 1, 2, and 10 µg mL^−1^). Columns show the mean ± SD of six independent replicates. Asterisks indicate significant differences: * *p* < 0.05; ** *p* < 0.01; *** *p* < 0.001.

Therefore, to evaluate the effects of LPS on colon cells, a preliminary study was conducted to determine the effect of different concentrations of this inflammatory agent. For this purpose, 2000 cells per well were seeded in 96-well plates and incubated for 24 h. After this time, different concentrations of LPS (1, 2, and 10 µg mL^−1^) were added, and the cells were cultured for 12, 24, and 48 h. After the corresponding time, cell viability was determined using the MTT assay. As shown in Figure 4, colon cell viability was significantly affected by both time and LPS concentration, but not by the interaction of both factors, as indicated by the results of the ANOVA analysis. Thus, cell viability increased progressively up to 48 h, reaching 100% viability at that point. Additionally, although the viability of cells exposed to different concentrations of LPS was slightly but significantly reduced after 12 h of exposure to LPS (1, 2, and 10 µg mL^−1^), it never dropped below 90%, indicating that the doses of LPS used were not cytotoxic.

### 2.3. Effect of GSLs-Enriched Extracts on Intracellular ROS Production in LPS-Stimulated CCD-18Co Myofibroblasts

**Figure 5:**
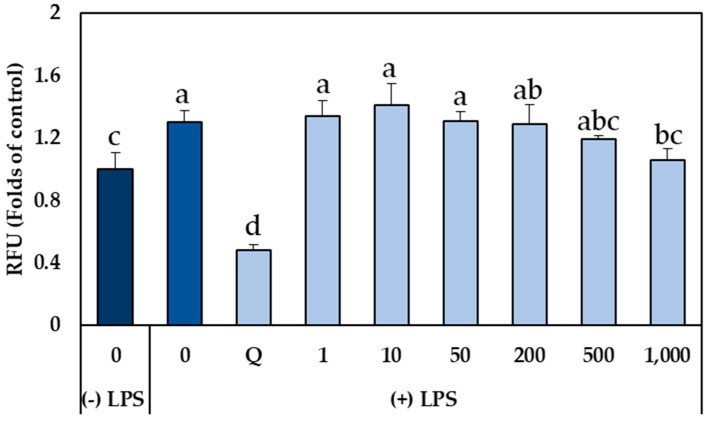
Relative fluorescence units (RFU, folds of control) of CCD-18Co cells due to intracellular ROS produced in the absence and presence of 2 μg mL^−1^ lipopolysaccharides (LPS) for 4 h subjected to 24 h pretreatment with different concentration of glucosinolates (GSLs)-enriched extract (0–1000 μg mL^−1^). Q: Quercetin. Data show the mean ± SD of six independent replicates. Different letters indicate significant differences according to Tukey’s test (*p*-value < 0.05).

The molecular mechanism of inflammatory injury can be attributed, at least partially, to the generation and release of ROS from activated neutrophils and macrophages. This overproduction of ROS can cause tissue injury by damaging membrane structures through lipid peroxidation, leading to an increase in the intestinal barrier’s permeability. Furthermore, ROS propagate inflammation by stimulating the release of cytokines that promote the recruitment of neutrophils and macrophages. Therefore, free radicals are key mediators that initiate or promote inflammation, and, consequently, attenuation of inflammation in terms of reduction in proinflammatory markers is directly related to disease prevention [30,31]. For these reasons, the elimination of ROS through the intake of antioxidant compounds in the diet has been considered as a strategy to improve the problems derived from the production of these compounds [2,9]. Thus, once the concentrations of GSLs-enriched extract obtained by PLE that did not inhibit cell growth of human colon myofibroblast CCD-18Co cells (1–1000 µg mL^−1^) were determined, and given that LPS did not significantly affect the cell viability of myofibroblasts, the potential cytoprotective effect of the GSLs-enriched extract on oxidative stress induced by LPS (2 µg mL^−1^) was evaluated to elucidate the underlying protective mechanisms in CCD-18Co cells (Figure 5). The results showed that treatment with LPS for 4 h significantly increased intracellular ROS levels in CCD-18Co cells up to 1.45 times compared to the control without LPS. However, in cells pretreated with GSLs-enriched extract for 24 h and exposed to LPS, intracellular ROS accumulation was significantly reduced as the extract concentration increased, reaching the basal level of cells not stimulated with LPS in the case of the highest extract concentration (1000 µg ml^−1^), which decreased ROS levels up to 0.71 times compared to LPS-stimulated cells (*p*-value < 0.05). These results correlate with the ABTS assay which shows a TEAC (Trolox Equivalent Antioxidant Capacity) value of 154.51 ± 13.26 µM TE g^−1^, indicating a relative capacity to neutralize ROS. In no cases did the pretreatment with the extract reduce the intracellular level of ROS as quercetin, which reduced ROS levels by 2.4 times compared to cells stimulated with LPS (*p*-value <0.05) (Figure 5).

### 2.4. Effect of GSLs-Enriched Extract on the Expression of Inflammation-Related Genes in LPS-Stimulated CCD-18Co Colon Myofibroblasts

**Figure 6:**
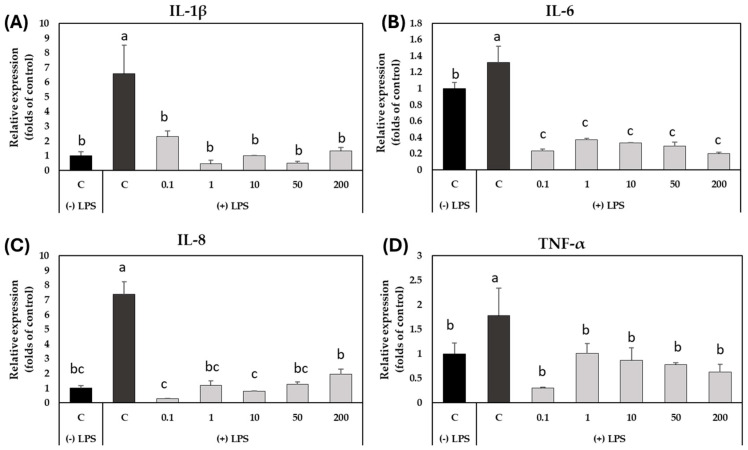
Variation in the relative expression (folds of the control) of the genes encoding proinflammatory cytokines (IL-1β (**A**), IL-6 (**B**), IL-8 (**C**), TNF-α (**D**)) in CCD-18Co cells pretreated with different concentrations of glucosinolates (GSLs)-enriched extract (0.1, 1, 10, 50, and 200 μg mL^−1^) after 8 h from exposure to 2 μg mL^−1^ lipopolysaccharides (LPSs). Values show the mean ± SD of three independent replicates. The LPS control (-) with reference value = 1 was used to normalize the relative expression levels of each gene. Transcript levels were calculated using the *GADPH* gene as reference gene. Different letters indicate significant differences according to Tukey’s test (*p*-value < 0.05).

To evaluate whether pretreatment with the GSLs-enriched extract could reduce oxidative stress caused by exposure to LPS in CCD-18Co cells, the expression profile of some genes encoding for proinflammatory cytokines (IL-1β, IL-6, IL-8, and TNF-α) was assessed by qRT-PCR (Figure 6). The ANOVA analysis showed that both the treatment with LPS and the concentration of the GSLs-enriched extract (*p*-value < 0.001) had a statistically significant influence on the expression of the genes analyzed.

The analysis of the expression profile of the gene corresponding to the proinflammatory cytokine IL-1β (Figure 6A) showed a maximum expression at 8 h due to the treatment with LPS; this increase was 6.6 times higher than the control. At that time, the pretreatment of the myofibroblasts with the GSLs-enriched extract significantly decreased the accumulation of transcripts compared to LPS-stimulated and unstimulated cells reaching the expression level of non-stimulated control cells (Figure 6A). Regarding the analysis of the relative expression of the *IL-6* gene (Figure 6B), a significant increase in the *IL-6* gene expression profile was also observed after 8 h of endotoxin stimulation, whereas after the pretreatment with the GSLs-enriched extract, the accumulation of transcripts was even lower than in the unstimulated control cells (Figure 6B).

Similarly, a significant increase in the accumulation of transcripts of the IL-8 proinflammatory cytokine (Figure 6C) was clearly observed in cells stimulated with LPS after 8 h of exposure; this was 7.7 times higher than the expression of the gene in non-stimulated cells. This increase in the expression level of the *IL-8* after the exposure to LPS was alleviated totally in GSLs-enriched extract pretreated cells at any concentration, as also occured with the cytokine IL-1β (Figure 6A).

Finally, the expression level of the gene that encodes for the cytokine TNF-α was also studied (Figure 6D). As shown in Figure 6, the qRT-PCR analysis revealed that stimulation with LPS endotoxin in the absence of the extract resulted in a maximum expression peak at 8 h after stimulation, which was 0.56 higher than the expression level in control cells. Once more the pretreatment with GSLs-enriched extract at any concentration was able to reduce the expression of this inflammation marker, in this case up to 2.97 times that of the expression in cells stimulated with LPS after 8 h.

## 3. Discussion

The results obtained demonstrated that broccoli GSLs can protect cellular components (such as DNA) from oxidative damage due to their ability to eliminate ROS, thereby playing an important role in the prevention of ROS-induced chronic intestinal diseases. The reduction in ROS observed after pretreatment of LPS-stimulated CCD-18Co cells with the broccoli GSLs-enriched extract agrees with previous research. In this context, several investigations have demonstrated the efficacy of adding compounds with antioxidant capacity to reduce the production of ROS in colon cells. For example, the study carried out by dos Santos et al. [9] demonstrated that açai extract, rich in polyphenols, partially reversed the generation of ROS induced by LPS in order of 0.53 times with respect to the control with LPS. Similarly, Angel-Morales et al. [2] evaluated the preventive potential of polyphenols extracted from red wine in LPS-stimulated human colon-derived CCD-18Co myofibroblast cells. Their results showed that when cells were stimulated with LPS, ROS levels increased 1.5-fold, but wine polyphenols prevented this induction, decreasing ROS levels by 0.58-fold, compared to ROS levels found in cells not stimulated with LPS.

Regarding broccoli, several studies have been conducted in this area. In this regard, Samuel et al. [32] observed a decrease in IL-8 levels in the serum of murine-fed steamed broccoli juice, probably due to the SFN content, which can inhibit NF-κB and thereby reduce the expression of this type of inflammatory mediator. On the other hand, Domínguez-Perles et al. [33] explored the use of broccoli-leaf extracts to enrich green tea infusions. The combination of bioactive compounds from both species (GSLs, hydroxycinnamic acids, flavonols, and catechins) resulted in increased antitumor activity in colon cancer cultures. The effect of cooking broccoli has also been studied in mice models of intestinal disease [34]. The results showed that, although the amount of plant myrosinase levels were lower when this vegetable was lightly cooked, the effects were similar to those observed when raw broccoli was consumed. Both raw and cooked broccoli consumption resulted in decreased disease activity, showing less reduction in colon length, less LPS leakage into the blood, and less severe colon lesions compared to those displayed by mice affected by the disease. Moreover, *IL-6* expression was also reduced in the groups of animals that consumed both raw and cooked broccoli. These results suggest the possible effective conversion of GSLs to their isothiocyanates by the gut microbiota.

Based on these studies and in view of the results obtained, GSLs-enriched extract obtained from broccoli by-products using PLE could be considered a potential ingredient for the development of new drugs for the treatment of IBD. Thus, the effects observed after analyzing the different inflammatory markers in response to LPS stimulation could be due to the high content of glucoraphanin (an aliphatic GSL precursor of the isothiocyanate SFN) and neoglucobrassicin, the major GSLs present in the optimized PLE extract. In this regard, Zhang and Wu [35] evaluated the ability of different concentrations of SFN (0.5–5 µM, equivalent to 0.09–0.9 µg mL^−1^) to protect Caco-2 cultures from LPS-induced injury. These concentrations are equivalent to the lower doses (0.1 and 1 µg mL^−1^) of the GSLs-enriched extract used in our study. Their results showed that SFN weakened the ability of LPS to induce production of inflammatory cytokines (IL-1β, IL-6, IL-8, and TNF-α) in a concentration-dependent manner, which agrees with our results, since these concentrations were able to significantly attenuate the expression of these cytokines. Likewise, studies conducted by Tian et al. [15] showed that dietary glucoraphanin supplementation in mice used as a colitis model reduced the expression of the inflammatory cytokines IL-1β, IL-18, and TNF-α, activated Nrf2, and alleviated oxidative stress. It was suggested that GSLs could protect the colon structure and prevent the inflammatory response of this disease. Similarly, indole-3-carbinol (an indole resulting from the hydrolysis of glucobrassicin) was able to significantly decrease the intestinal expression of NF-κB and several inflammatory factors such as TNF-α, IL-1β, IL-6, and IL-8 in mice with colitis [36]. Furthermore, numerous studies have examined the potential of SFN, the most promising isothiocyanate, specifically on its effect on intestinal health. These studies suggest that SFN’s mechanism of action involves the translocation of Nrf2 to the cell nucleus, where it binds to antioxidant response elements (AREs) in the promoter region of several phase II detoxification enzymes [37,38]. This compound has been shown to dose-dependently inhibit the production of inflammatory cytokines such as TNF-α, IL-1β, and IL-6 in colon cancer cell cultures [39]. Additionally, it has also been demonstrated that SFN can eradicate *Helicobacter pylori* strains that cause intestinal damage such as gastritis and peptic ulcers [40], as well as inhibiting the proliferation of HCT-116 human colorectal cancer cells [41]. Wei et al. [42] also studied the use of SFN as a treatment for intestinal injuries caused by antitumor treatments. Their results showed that SFN supplementation in the diet of mice treated with 5-fluorouracil improved the intestinal injury caused by the treatment, alleviated weight loss, repaired the structure of the damaged epithelium in both the jejunum and the colon, reduced intestinal inflammation (by decreasing the expression of NF-kB and increasing that of Nrf2), and healed intestinal permeability.

The results demonstrated that the exposure of colon myofibroblasts to LPS resulted in intracellular ROS accumulation and overexpression of inflammatory-related genes. In addition, pretreatment of colon cells with GSLs-enriched extract was able to mitigate the oxidative and inflammatory response. However, even though some molecular mechanisms have been identified in vivo and clinical trials [43], there are still multiple challenges and gaps in knowledge for studying their functionality to validate their efficacy in humans. These include factors such as the amount and quality of the extract consumed, its bioavailability, metabolism, and the biological activity of GSLs and their hydrolysis products in the treatment and/or prevention of IBD. To address these issues, further in vitro research should be performed to corroborate the impact of this GSLs-enriched extract as a potential ingredient in formulations for the amelioration of IBD.

## 4. Materials and Methods

### 4.1. Chemicals and Reagents

Glucoraphanin, glucoiberin, glucoalyssin, glucoerucin, glucobrassicin, and gluconasturtiin (assay > 97%) were from Phytoplan Diehm & Neuberger GmbH (Heidelberg, Germany). Ethanol and dimethyl sulfoxide (DMSO) were of analytical grade. Eagle’s Minimal Essential Medium (EMEM) was from Invitrogen (Carlsbad, CA, USA). Double-deionized water with conductivity lower than 18.2 MV was obtained with a Milli-Q system (Millipore, Bedford, MA, USA) and ethanol (purity ≥ 99.9%) was used as co-solvent for the PLE and was provided by VWR chemicals (Radnor, PA, USA). Ottawa sand was provided by Fisher Scientific (Leicester, UK). LPS from *Escherichia coli* O111:B4 was from Sigma–Aldrich (Taufkirchen, Germany).

### 4.2. Sample Preparation

Broccoli (*Brassica oleracea* L. var. italica, cv Naxos) by-products (stems and leaves) were provided by Agrícola Santa Eulalia S.L (Murcia, Spain). By-products were cut and ground using a Comitrol^®^ Processor Model 1700 Urschell (Chesterton, IN, USA) and then they were dried in a climatic chamber at 55 °C for 24 h until reaching a final moisture content of 4%. Finally, dried samples were finely ground to obtain a homogeneous powder as previously reported by Borja-Martinez et al. [20]. For further analysis, the dried broccoli by-products were stored at room temperature and in darkness until use.

### 4.3. Extraction of GSLs Using Pressurized Liquids

GSLs extraction was performed using a Dionex ASE 350 pressurized liquid extractor (Dionex Corp., Sunnyvale, CA, USA). Briefly, 2 g of dried broccoli by-products were homogeneously mixed with 4 g of sand, and the samples were loaded into 33 mL stainless steel extraction cells. A cellulose filter was placed in each lid beforehand to prevent suspended particles from passing into the collection vial. Next, these cells were introduced into an oven, sealed, and subjected to the predetermined temperature and pressure conditions for each experiment. After completing the extraction cycles, the obtained extracts were immediately cooled on ice until reaching a temperature of 20 to 25 °C. Subsequently, the extracts were filtered through a 0.22 µm cellulose filter and centrifuged at 12,000 rpm (Sorvall ST 16 R, Thermo Scientific, Leicestershire, UK) for 15 min at 4 °C. Finally, the supernatants were evaporated under vacuum (13 kPa) at 35 °C using a Speed-Vac Savant SC250EXP (Thermo Scientific, Leicestershire, UK). The resulting extracts were stored at −20 °C, protected from light, until further analysis.

To optimize the recovery of GSLs, a RSM with a CCD 2^3^ (3 independent variables and two levels) was applied using the Statgraphics Centurion XVI software. As independent variables, temperature (53, 70, 128, 185, and 200 °C), ethanol percentage (5, 15, 50, 85, and 95%) and extraction time (3, 5, 12,5, 20, and 22 min) were set. The response variable was the GSLs content (μg g^−1^ of extract). In total, 16 experiments were carried out in randomized order, from which, the optimal extraction conditions were established to obtain the GSLs-enriched extract by PLE. The extraction yield (EY) was calculated as the percentage of total mass of dry extract per gram of extracted raw material used in the extraction procedure (EY = (amount of dry extract/amount of raw material) × 100).

### 4.4. Conventional Extraction of GSLs

GSLs were extracted with methanol–water (70:30 *v*/*v*) according to Sánchez-Pujante et al. [44] from 200 mg of dried and homogenized broccoli by-products. To avoid the GSLs degradation by myrosinase, samples were heated at 73 °C for 20 min. After that, the samples were cooled in an ice bath for 5 min and centrifuged (10,160× *g* for 15 min at 4 °C). The supernatant was collected, and the pellet was extracted again. The supernatant was evaporated under vacuum. Finally, the extract was resuspended in 2 mL of deionized water, filtered (0.22 μm PVDF filters, Millipore, Germany), and stored at −20 °C for further analysis.

### 4.5. Quantification of GSLs in PLE and CE Extracts

Identification and quantification of GSLs was carried out using a HPLC system (Agilent Series 1200, Agilent Technologies, Santa Clara, CA, USA)

USA coupled to a triple quadrupole mass spectrometer (MS/MS) with an electrospray ionization (ESI) source operating in negative mode, following the methodology described by Flores et al. [45]. Chromatographic separation was performed on a Luna C18 column (250 mm × 46 mm, 5 μm particle size; Phenomenex, Macclesfield, UK) with a C18-ODS precolumn (4 mm × 30 mm) (Security Guard, Phenomenex, Macclesfield, UK) at room temperature. The mobile phase employed was 0.1% trifluoroacetic acid in water (A) and 0.1% trifluoroacetic acid in acetonitrile (B) with the following gradient: 0 min, 100% A; 5 min, 100% solvent A; 15 min, 83% A; 17 min, 83% A; 22 min, 75% A; 30 min, 65% A; 35 min, 50% A; and 45 min, 1% A and 50 min, 100% A. The flow rate was 0.7 mL min^−1^ and the injection volume was 20 μL. GSLs identification was carried out using a co-chromatography with authentic standard, absorbance spectra, and fragmentation patterns obtained by mass spectrometry as previously described by Tian et al. [46]. Glucoraphanin, glucoiberin, glucoalyssin, glucoerucin, glucobrassicin, and gluconasturtiin were quantified with respect to their corresponding standards, whereas 4-hydroxyglucobrassicin, 4-metoxyglucobrassicin, and neoglucobrassicin were quantified with respect to glucobrassicin. The MS/MS transitions were *m*/*z* 436 → 97 for glucoraphanin, 422 → 97 for glucoiberin and gluconasturtiin, 450 → 97 for glucoalyssin, 420 → 97 for glucoerucin, 447 → 97 for glucobrassicin, 463 → 97 for 4-hydroxyglucobrassicin, and 477 → 97 for 4-metoxyglucobrassicin and neoglucobrassicin. Thus, due to the deprotonated molecules ([M − H]^−^) of intact GSLs producing a characteristic fragment of *m*/*z* 97 ([SO3H]^−^), each GSL was quantified based on the transition [M − H]^−^ → *m*/*z* 97 (Figure S1). The calibration curves of these external standards were as follows: glucoraphanin (y = 4130.1x − 19.3; *R*^2^ = 0.999); glucoiberin (y = 5984.8x + 38.9; *R*^2^ = 0.999); glucoalyssin (y = 8207.9x + 122.4; *R*^2^ = 0.999); glucoerucin (y = 9629.7 − 1878.6; *R*^2^ = 0.997); glucobrassicin (y = 6749.5x − 460.4; *R*^2^ = 0.999); and gluconasturtiin (y = 11,180x − 1414.3; *R*^2^ = 0.998). The range of each standard curve was 0.07–25 µg mL^−1^.

### 4.6. CCD-18Co Cell Culture and Anti-Inflammatory Assays

Human colon-derived CCD-18Co (ATCC^®^ CRL1459™) myofibroblast cells were obtained from ATCC (Manassas, VA, USA). The cells were maintained in Eagle’s Minimum Essential Medium (EMEM) supplemented with 10% fetal bovine serum and cultured in a humidified incubator containing 5% CO_2_ at 37 °C. For cell culture assays, CCD-18Co cells were seeded on a 96-well microtiter plate at a density of 2 × 10^3^ cells per well for 24 h to allow cells to stabilize and attach onto the bottom of the well. Thereafter, the GSLs-enriched extracts and/or the LPS were added to the cells at concentrations ranging from 0.1 to 2000 µg mL^−1^ and from 1 to 10 µg mL^−1^, respectively.

#### 4.6.1. Cell Viability Assay

The cell viability of CCD-18Co cells treated with GSLs-enriched extract and/or LPS was evaluated using the colorimetric MTT [3-(4,5-dimethylthiazol-2-yl)-2,5-diphenyltetrazoliumbromide] assay. For this, after the exposure of the CCD-18Co cells to the extract and/or LPS, the culture medium was removed and replaced with 200 μL of a filtered 1 mg mL^−1^ MTT solution (in serum-free DMEM), and then the cells were incubated in darkness at 37 °C/5% CO_2_ for 4 h. After that, the MTT solution was removed and replaced with 100 μL of dimethylsulfoxide to dissolve the violet formazan crystals. After 5 min of gentle shaking, the absorbance was measured spectrophotometrically at 570 and 690 nm using a microplate reader FLUOstar Omega (BMG Labtech, Ortenberg, Germany). Cell viability was calculated as the percentage of viable cells compared to the control.

#### 4.6.2. In Vitro Antioxidant Activities

The antioxidant capacity of the GSLs-enriched extract obtained from the broccoli by-products was determined as previously reported by Borja-Martínez et al. [20], using the Trolox Equivalent Antioxidant Capacity (TEAC) method or ABTS method. The ability of the extracts obtained through PLE to neutralize free radicals was evaluated by measuring their effect on the stable ABTS^●+^ radical. The interaction of the samples with ABTS^●+^ was analyzed spectrophotometrically at 414 nm, following the methodology described by Escribano et al. [47]. To generate the ABTS^●+^ radical, a 20 mM ABTS stock solution was prepared in the presence of peroxidase (1 mg/mL of commercially available horseradish peroxidase type VI from Sigma), hydrogen peroxide, and a 0.2 M sodium acetate buffer at pH 5.0. The reactions were conducted in a 0.2 M sodium phosphate buffer with a pH of 7.0. Absorbance readings were taken at 414 nm using 1.5 mL plastic cuvettes at the start of the experiment and after 24 h of incubation at 20 °C in darkness, using a Jasco V-730 spectrophotometer (Easton, MD, USA). All assays were performed in triplicate. The TEAC values were expressed as µM of Trolox equivalents per gram of extract.

#### 4.6.3. Determination of Intracellular ROS Production

Intracellular ROS levels were evaluated by using the fluorescent probe 2′,7′dichlorodihydrofluorescein diacetate (DCFH-DA) (Sigma-Aldrich, Hamburg, Germany). For this, CCD-18Co cells were seeded on a 96-well plate (3 × 10^3^ cells per well) in EMEM complete medium for 24 h to allow for attachment, and then cells were pre-treated with GSLs-enriched extract ranging from 1 to 1000 µg mL^−1^ for 24 h in darkness at 37 °C for 30 min. Then, the culture medium with the extracts was discarded and replaced with a solution of 2 μg mL^−1^ LPS to generate ROS for 4 h, followed by washing out the cells twice using phosphate-buffered saline solution. After that, the cells were stained in situ with 100 μL of 10 μM DCFH and further incubated for 30 min under standard culture conditions. Afterwards, the cells were washed twice with PBS and fluorescence signal was monitored at 520 nm (emission) and 485 nm (excitation) using a FLUOstar Omega plate reader (BMG Labtech Inc., Durhan, NC, USA). Relative fluorescence units (RFU) were analyzed using MARS Software version 5.3 and normalized to control cells (non-treated with LPS or GSLs-enriched extracts).

#### 4.6.4. RNA Isolation and Quantitative Real-Time RT-PCR

Total RNA was isolated from CCD-18Co cells using the RNeasy Mini Kit (Qiagen, Hilden, Germany) according to the manufacturer’s specification. For this, myofibroblasts were previously seeded into 6-well plates at a density of 9 × 10^4^ cells per well in complete EMEM. After 48 h of, the cells were pre-treated with different concentrations of GSLs-PLE extracts ranging from 0.1 to 200 μg mL^−1^ for 24 h. Then, CCD-18Co cells were exposed to 2 μg mL^−1^ LPS for 4 h, as described above. At 8 h after LPS exposure, the culture medium was discarded, and the cells were lysed to extract RNA using the RNEasy MiniKit (Qiagen, Hilden, Germany), following the manufacturer’s instructions. cDNA was synthetized from 1 μg of purified RNA using the RevertAid First Strand cDNA Synthesis Kit (Thermo Fischer Scientific, Waltham, MA, USA) according to the manufacturer’s protocol.

Real-time PCR analysis was conducted using an Applied Biosystems QuantStudio™ 5 Flex Real-Time PCR system (Thermo Fisher Scientific, Waltham, MA, USA) in a total volume of 10 μL. The reaction mixture included 5 μL of 2X Power SYBR Green PCR Master Mix (Applied Biosystems, Carlsbad, CA, USA), 51 nM of both forward and reverse gene-specific primers previously reported by Borja-Martínez et al. [48], and 33 ng of cDNA template. The PCR cycling conditions consisted of an initial step of 2 min at 50 °C, followed by 10 min at 95 °C, and then 40 cycles of 15 s at 95 °C and 1 min at 60 °C. Gene expression was normalized using glyceraldehyde-3-phosphate dehydrogenase (*GAPDH*) as the reference gene. All experiments were conducted in triplicate.

### 4.7. Statistical Analysis

In addition to the statistical analysis performed to optimize PLE, data were analyzed by two-way analysis of variance (ANOVA) followed by Tukey’s test to examine the significance of the observed differences using the SPSS package (SPSS Inc., Chicago, IL, USA) version 22, and *p*-values <0.05 were considered as statistically significant.

## 5. Conclusions

An efficient extraction procedure of GSLs from broccoli by-products based on PLE (using ethanol/water as solvent) has been developed and optimized, with the optimal values of the PLE conditions for the GSLs extraction being 53 °C, 8 min, and 83% ethanol, thus demonstrating the efficiency of PLE as an environmentally sustainable alternative to extract GSLs from broccoli by-products. Furthermore, GSLs-enriched extract obtained by PLE possesses anti-inflammatory properties in LPS-stimulated CCD-18Co colon cells and has the potential to attenuate the production of some proinflammatory markers (IL-1β, IL-6, IL-8, and TNF-α), in addition to being a potential antioxidant, capable of reversing the intracellular accumulation of ROS. However, complementary studies are required to corroborate this application so that it can be considered as a promising anti-inflammatory dietary component.

## Figures and Tables

**Table 1:** Central composite design 2^3^ (CCD) independent and response variable values.

Condition	Temperature (°C)	Ethanol (%)	Time (min)	Response Variable * (µg GSLs g^−1^ Extract)	EY (%)
PLE-1	70	15	20	1926 ± 59	37
PLE-2	128	50	22	1896 ± 62	38
PLE-3	70	85	5	2565 ± 81	27
PLE-4	128	50	12.5	1701 ± 56	37
PLE-5	70	15	5	1820 ± 57	38
PLE-6	128	50	12.5	1839 ± 59	36
PLE-7	185	15	5	531 ± 20	56
PLE-8	128	50	3	1661 ± 55	36
PLE-9	200	50	12.5	335 ± 14	40
PLE-10	128	95	12.5	977 ± 29	22
PLE-11	185	15	20	696 ± 25	52
PLE-12	128	5	12.5	1145 ± 35	45
PLE-13	185	85	5	155 ± 5	38
PLE-14	185	85	20	194 ± 8	41
PLE-15	70	85	20	3649 ± 116	27
PLE-16	53	50	12.5	2930 ± 94	32

* Response variable is given as the mean ± standard deviation of four replicates. EY, extraction yield.

**Table 2:** Analysis of variance (ANOVA) and fitting parameters of the proposed CCD 2^3^ model. Significant coefficients (* *p* < 0.05). SS = sum of squares; Df = degrees of freedom; MS = mean square; R^2^ = quadratic correlation coefficient.

Source	Y = Glucosinolate Content				
	SS	Df	MS	F-Ratio	*p*-Value
X_1_: Temperature	1.21 × 10^7^	1	1.21 × 10^7^	131.88	0.0000 *
X_2_: Ethanol	166,424	1	166,424	1.81	0.2209
X_1_ X_2_	1.40 × 10^6^	1	1.40 × 10^6^	15.19	0.0059 *
X_2_ X_2_	680,824	1	680,824	7.39	0.0298 *
Lack-of-fit	234,659	4	58,664.7	0.64	0.6527
Pure error	644,881	7	92,125.8		
Total (corr.)	1.53 × 10^7^	15			
*R* ^2^	0.942				

**Table 3:** Comparison between experimental results and predicted values by the model generated for GSLs content (μg g^−1^) from the pressurized liquid extraction (PLE). CV = coefficient of variance.

Condition	Theoretical	Experimental	CV (%)
PLE-1	1895	1926 ± 59	0.6
PLE-2	1741	1896 ± 62	3.0
PLE-3	2974	2565 ± 81	5.2
PLE-4	1741	1701 ± 56	0.8
PLE-5	1895	1820 ± 57	1.4
PLE-6	1741	1839 ± 59	1.9
PLE-7	659	531 ± 20	7.6
PLE-8	1741	1661 ± 55	1.7
PLE-9	444	335 ± 14	9.8
PLE-10	1310	977 ± 29	10.3
PLE-11	659	696 ± 25	1.9
PLE-12	1008	1145 ± 35	4.5
PLE-13	65	155 ± 5	28.9
PLE-14	65	194 ± 8	35.3
PLE-15	2974	3649 ± 116	7.2
PLE-16	3093	2930 ± 94	1.9
Optimal conditions	3405	4530 ± 132	10

**Table 4:** Total glucosinolates (GSLs; μg g^−1^ extract) obtained from broccoli by-products under optimal pressurized liquid extraction (PLE) conditions (PLE-OP) and conventional extraction (CE). EY: extraction yield. n.d.: undetected compound.

Glucosinolates		PLE-OP (μg g^−1^ Extract)	CE (μg g^−1^ Extract)
Alifatic	Glucoiberin	6.9 ± 0.4	8 ± 1
	Glucoraphanin	2144 ± 101	4888 ± 961
	Gluconapin	0.6 ± 0.04	n.d.
	Glucoalisin	14 ± 1	27 ± 5
	Glucoerucin	380 ± 10	919 ± 177
	Glucoraphenin	n.d.	4 ± 2
Indolic	Glucobrassicin	216 ± 12	471 ± 88
	Neoglucobrassicin	1497 ± 141	2116 ± 370
	4-Hydroxyglucobrassicin	22 ± 1	82 ± 17
	4-Metoxyglucobrasicin	130 ± 11	258 ± 39
Aromatic	Glucotropaeolin	n.d.	8 ± 1
	Gluconasturtiin	120 ± 12	234 ± 50
	TOTAL	4530 ± 132	9015 ± 449
	EY (%)	21	31

## Data Availability

The original contributions presented in this study are included in the article and Supplementary Materials. Further inquiries can be directed to the corresponding author.

## Supplementary Materials

The following supporting information can be downloaded at: https://www.mdpi.com/article/10.3390/plants14111700/s1, Figure S1. (A) Total ion chromatogram of the optimized pressurized liquid (PLE) and conventional (CE) extracts derived from broccoli by-products. (B) Retention time (R_t_), basic structure (R: side chain), Structure of R, common name of glucosinolates, precursor ion [M − H]^−^ and fragment ions data for the identified compounds; Table S1: title.
