# Supplementary material for: Glucosinolates from Broccoli By-Products Obtained by Pressurized Liquid Extraction Exert Anti-Inflammatory Activity on Non-Malignant Colonic Myofibroblasts
Source: Plants (Basel). 2025 Jun 3;14(11):1700. doi: 10.3390/plants14111700 (PMC12157804; doi:10.3390/plants14111700)
Supplement: Supplementary file 1 [file plants-14-01700-s001.zip › plants-3645274-supplementary.pdf]

A)

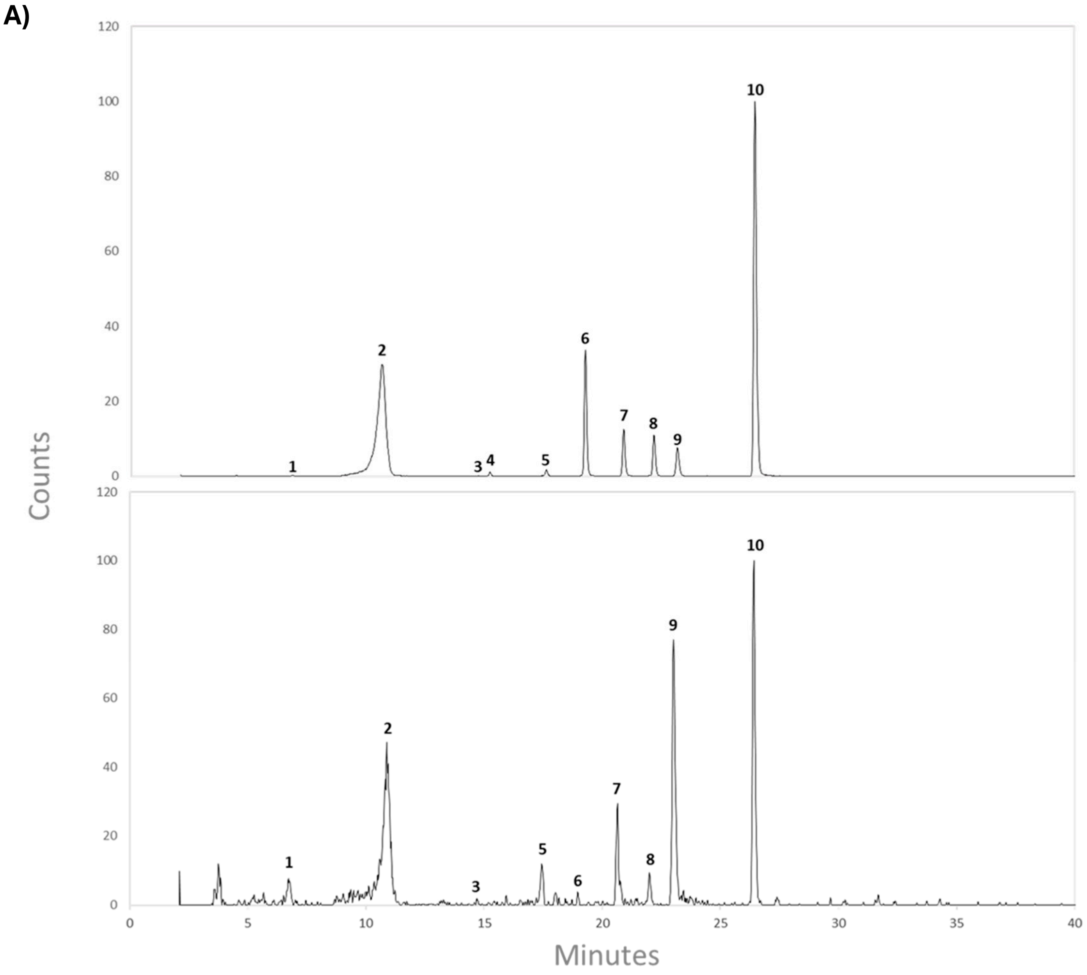

B)

Peak	R_t (min)	Basic structure	Structure of R	Common name	Precursor ion $[M-H]^-$, m/z	Fragment ions m/z
1	7.05		H_3C-SO-$(CH_2)_3$-	Glucoiberin	422	358,97
2	11.77		H_3C-SO-$(CH_2)_4$-	Glucoraphanin	436	372,97
3	14.74		H_3C-SO-$(CH_2)_5$-	Glucoalyssin	450	386,97
4	15.51		CH_2=CH-CH_2-CH_2-	Gluconapin	372	259,97
5	17.23		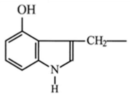	4-Hidroxyglucobrassicin	463	285,267,97
6	19.05		CH_3-S-CH_2-CH_2-CH_2-CH_2-	Glucoerucin	420	259,97
7	20.6	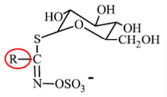	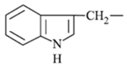	Glucobrassicin	447	259,97
8	22.02		$C_6H_5CH_2CH_2$–	Gluconasturtiin	422	259,97
9	22.83		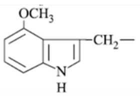	4-Methoxyglucobrassicin	477	259,241,97
10	26.25		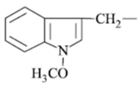	Neoglucobrassicin	477	447,446,259,97

Supplementary Figure 1. A) Total ion chromatogram of the optimized pressurized liquid (PLE) and conventional (CE) extracts derived from broccoli by-products. **B)** Retention time **(**R_t), basic structure (R: side chain), Structure of R, common name of glucosinolates, precursor ion [M-H]- and fragment ions data for the identified compounds.
